# Chronic Fluoxetine Treatment Induces Maturation-Compatible Changes in the Dendritic Arbor and in Synaptic Responses in the Auditory Cortex

**DOI:** 10.3389/fphar.2019.00804

**Published:** 2019-07-17

**Authors:** Estibaliz Ampuero, Mauricio Cerda, Steffen Härtel, Francisco Javier Rubio, Solange Massa, Paula Cubillos, Lorena Abarzúa-Catalán, Rodrigo Sandoval, Albert M. Galaburda, Ursula Wyneken

**Affiliations:** ^1^Instituto de Ciencias Biomédicas, Facultad de Ciencias de la Salud, Universidad Autónoma de Chile, Santiago, Chile; ^2^SCIAN-Lab, CIMT, Biomedical Neuroscience Institute (BNI), ICBM, Faculty of Medicine, University of Chile, Santiago, Chile; ^3^Centro Nacional de Sistemas de Información en Salud (CENS), Faculty of Medicine, University of Chile, Santiago, Chile; ^4^Laboratorio de Neurociencias, Universidad de los Andes, Santiago, Chile; ^5^Departamento de Ciencias Biomédicas, Facultad de Medicina, Universidad Católica del Norte, Coquimbo, Chile; ^6^Department of Neurology, Harvard Medical School and Beth Israel Deaconess Medical Center, Boston, MA, United States

**Keywords:** dendritic architecture, auditory cortex, neuronal segmentation, antidepressant, NMDA receptors

## Abstract

Fluoxetine is a selective serotonin reuptake inhibitor (SSRI) used to treat mood and anxiety disorders. Chronic treatment with this antidepressant drug is thought to favor functional recovery by promoting structural and molecular changes in several forebrain areas. At the synaptic level, chronic fluoxetine induces an increased size and density of dendritic spines and an increased ratio of GluN2A over GluN2B N-methyl-D-aspartate (NMDA) receptor subunits. The “maturation”-promoting molecular changes observed after chronic fluoxetine should also induce structural remodeling of the neuronal dendritic arbor and changes in the synaptic responses. We treated adult rats with fluoxetine (0.7 mg/kg i.p. for 28 days) and performed a morphometric analysis using Golgi stain in limbic and nonlimbic cortical areas. Then, we focused especially on the auditory cortex, where we evaluated the dendritic morphology of pyramidal neurons using a 3-dimensional reconstruction of neurons expressing mRFP after *in utero* electroporation. With both methodologies, a shortening and decreased complexity of the dendritic arbors was observed, which is compatible with an increased GluN2A over GluN2B ratio. Recordings of extracellular excitatory postsynaptic potentials in the auditory cortex revealed an increased synaptic response after fluoxetine and were consistent with an enrichment of GluN2A-containing NMDA receptors. Our results confirm that fluoxetine favors maturation and refinement of extensive cortical networks, including the auditory cortex. The fluoxetine-induced receptor switch may decrease GluN2B-dependent toxicity and thus could be applied in the future to treat neurodegenerative brain disorders characterized by glutamate toxicity and/or by an aberrant network connectivity.

## Introduction

Selective serotonin reuptake inhibitors (SSRI) such as fluoxetine, used in chronic antidepressant treatment, induce adaptive rearrangements in the central nervous system that are not fully characterized. At the structural level, fluoxetine induces an enlargement of dendritic spines and increased spine density in several forebrain regions (Hajszan et al., 2005; Guirado et al., 2009; Ampuero et al., 2010; Kitahara et al., 2016). In adult rats, this is associated with a switch of synaptic glutamate receptor subunits favoring an enrichment of NMDA receptors (NMDA-Rs) containing preferentially GluN2A over GluN2B subunits (Ampuero et al., 2010; Rubio et al., 2013; Beshara et al., 2016), a switch consistent with synaptic maturation (Paoletti et al., 2013). NMDA-Rs are hetero-tetramers composed obligatorily of two GluN1 and two GluN2 subunits, which, in the majority of forebrain neurons, correspond to GluN2A and GluN2B. During development, GluN2B expression decreases dramatically. This goes along with increases in GluN2A-containing NMDA-Rs, which are mainly located in mature synapses and mediate cell survival. Conversely, GluN2B-containing NMDA-Rs are located in less stable (i.e., plastic) synapses, as well as at extrasynaptic sites, where they favor cell death (Paoletti et al., 2013). The developmental switch towards higher GluN2A expression, as well as experimental introduction of GluN2A subunits, produces a reduction in the dendritic length and branching that depends on different, C-terminal specific, intracellular signaling capacities (Kannangara et al., 2014; Bustos et al., 2017; Keith et al., 2019). In contrast, introduction of GluN2B subunits increase the number of dendritic branches and dendritic length (Sepulveda et al., 2010; Bustos et al., 2014). Considering the widespread increase in GluN2A subunits following fluoxetine, we examined structural and synaptic adaptations in cortical areas that are not directly involved in the modulation of mood, such as sensory (including auditory) and motor cortices, which are impaired in depression and might be affected by fluoxetine treatment (Zwanzger et al., 2012; Frodl, 2017; Yin et al., 2018). We specifically evaluated the dendritic architecture and the synaptic properties of pyramidal neurons in the auditory cortex after fluoxetine treatment, an area that is disturbed in depression (Zweerings et al., 2019). Our results indicate that fluoxetine induces structural and functional adaptations compatible with increased network maturation. These properties of fluoxetine could be useful in the treatment of diseases characterized by altered GluN2B over GluN2A ratios and the associated growth of aberrant dendritic arbors and/or of diseases characterized by NMDA-R-dependent toxicity due to GluN2B-dependent calcium overload.

## Materials and Methods

### Materials

All chemical reagents were purchased from Sigma (St Louis, MO), unless otherwise stated.

### Animals

Male Sprague–Dawley rats were housed in groups of three to four animals in their home cages and kept under a 12-h light/dark cycle (light on at 7 am) cycle at 22 ± 1°C. Food and water were available *ad libitum*. At the beginning of the experiment, rats were 78 days old, and their average weight was 266.5 ± 42 g (control group) and 269 ± 40 g (fluoxetine group). At the end of the treatment period, rats were 106 days old and weighted 392 ± 51 and 372 ± 53 g on average, respectively.

As in our previous studies, fluoxetine at a dose of 0.7 mg/kg (Ely-Lilly Co., Indianapolis, USA) or saline solution (control) was administered by intraperitoneal (i.p.) injection once daily between 9 and 10 am for 28 days. This dose induces increased BDNF expression and TrkB-dependent signaling at synapses, as well as remodeling glutamate neurotransmission and GluN2A over GluN2B switch of NMDA-R subunits (Wyneken et al., 2006; Ampuero et al., 2010; Rubio et al., 2013). This study was carried out in accordance with the Guide for the Care and the Use of Laboratory Animals from the National Institutes of Health (Ed 8; http://grants.nih.gov/grants/olaw/Guide-for-the-Care-and-Use-of-Laboratory-Animals.pdf) and the Bioethical Guidelines for the Use of Laboratory Animals from Universidad de los Andes Bioethical Committee. The protocol was supervised by the Universidad de los Andes Bioethical Committee.

### Golgi Staining and Morphometric Analysis

To perform Golgi staining, the adult rats were sacrificed under ketamine (50 mg/kg) and xylazine (5 mg/kg) anesthesia, 24 h following the last fluoxetine or saline dose (*n* = 10–11 per group). The rats were perfused intracardially with saline solution followed by 300 ml of 4% buffered paraformaldehyde solution. Immediately after perfusion, the brains were removed and processed for Golgi staining using GolgiStainTM kit as previously described (Ampuero et al., 2007).

Coronal sectioning at 150 µm was performed to analyze dendritic morphology. The morphometric analysis of pyramidal neurons was restricted to stereotaxic coordinates (in mm): for the prelimbic (PrL) cortex to: interaural 13.20 and Bregma −4.20, for the secondary motor cortex (M2) to: interaural 5.86 and Bregma −3.14, for retrosplenial granular b cortex (RSGb) to: interaural 5.20 and Bregma −3.8 mm and for primary auditory cortex (AUD1) to: interaural 4.70 and Bregma −4.30 (Paxinos and Watson, 1998).

Golgi-stained images were obtained with a Nikon Eclipse TE2000-U inverted epifluorescence microscope with a Plan Fluor 20x/1.25 numeric aperture attached to a cooled monochrome camera DS-2MBWc.

Pyramidal neurons were defined by the presence of basilar dendrites, a distinctive, single apical dendrite, and dendritic spines (Spruston, 2008). The observer, blind to experimental conditions, selected randomly at least 10 neurons from each animal that fulfilled the following selection criteria: 1) absence of truncated dendrites, 2) consistent and dark impregnation along the entire dendritic field, and 3) spatial separation from neighboring impregnated neurons to avoid overlap. Camera lucida tracings from selected neurons (500×, BH-2, Olympus Co., Tokyo, Japan) were performed and then scanned (eight-bit grayscale TIFF images with 1,200 dpi resolution; EPSON ES-1000C) along with a calibrated scale for subsequent computerized image analysis. For morphometric analysis of digitized images, we used Image J software (National Institutes of Health). In each selected neuron, the total, basal, and apical dendritic lengths were determined.

### *In Utero* Electroporation

For *in utero* electroporation, five pregnant young female rats at embryonic day 16.5 were anesthetized with xylazine (5 mg/kg) and ketamine (50 mg/kg). After shaving the rat’s belly at the location of the desired incision and disinfecting the abdominal skin with povidone solution, an approximately 2-cm midline skin incision was made followed by another incision along the linea alba. Then, both uterine horns were drawn out through the incision. To inject the plasmid, glass capillaries (pulled with the P97 Pipet Puller, Sutter Instruments, USA) were filled with the plasmid solution (1 μg/μl), mixed with Fast Green (<0.75 mg/ml, Sigma Aldrich, USA), and delivered into the left lateral ventricle with a pressure Pico pump (PV830, World Precision Instruments, USA). The plasmid containing the CAG promoter (i.e., driven by the strong ubiquitous promoter cytomegalovirus early enhancer and chicken beta actin) and the monomeric red fluorescent protein (mRFP) sequence, kindly shared by Joseph LoTurco, was used (LoTurco et al., 2009) (**Supplementary Figure 1**).

Electric pulses were applied with an electroporation system built with a power supply, a resistor, pulse/charge power switch, capacitor, and a foot pedal for providing the pulse. To introduce the plasmid into a larger region of the cortex, the cathode was placed on the location corresponding to the left temporal lobe, and the anode was placed on the location corresponding to the right temporal lobe. Voltage pulses were adjusted to the developing stage of the fetuses (65 mV for E16). Only one pulse lasting 3 s was applied. The embryos closest to the cervix were not injected nor electroporated because the possible death of these animals could block the delivery. After the procedure was completed, the two horns were reinserted into the abdominal cavity with an additional 5 ml of isotonic saline solution, and then, the abdominal wall and skin were closed with nylon and silk sutures, respectively. After surgery, the rats gave birth normally. Only male pups (*n* = 18) underwent further induction and treatment to circumvent hormonal differences influencing the results. At 78 days of age rats began fluoxetine treatment. Overall, the effectiveness of the electroporation procedure was 41%, and from these, 30% expressed mRFP in the primary auditory cortex (AUD1) resulting in six rats for 3-dimensional analysis.

### 3D Analysis

#### Image Acquisition

The images from mRFP-electroporated animals were obtained using a Leica LSI Macro-Zoom confocal laser scanning microscope with a 5× air objective (NA = 0.12) plus optical zoom 1.7×, excitation with solid state laser at 488 nm, and spectral detection. TIFF images (8 bits) of 1,024 × 1,024 pixels were acquired with 0.1 μm between *z*-sections and then projected into one 2D image (maximum intensity projection). To obtain a broad field of acquisition, the mosaic function in LAS-AF software (Leica Microsystems, Bannockburn, IL, USA) was used. At least 50–70 focal planes of 0.1 µm were obtained.

#### Image Segmentation

Semi-automatic segmentation was applied to the neuron stacks. The automatic part was implemented in FIJI software (Schindelin et al., 2012), sequentially using a normalization step to reduce variability among samples (using the full histogram), applying an anisotropic diffusion filter (parameters 0.1 and 0.99) to smooth neuron processes without blurring edges, highlighting elongated areas associated with processes with the Frangi filter (default parameters), and finally with a manual threshold to binarize the stack. A manual check verified that close neurons were at least separated by 1 voxel and the 3D connectivity of the object, so a simple 3D binary object was in the stack.

#### Skeleton Estimation

The segmented neuron was simplified into a graph, or skeleton, where segments represent processes, and branching points represent nodes. The skeleton estimation algorithm used as input a surface triangle-mesh geometrical representation that was computed in IDL (Lorensen and Cline, 1987). In brief, the skeleton computation is an optimization algorithm that searches for the best graph making a balance between two terms: a good representation of the original volume and use of as few segments as possible as proposed initially by Au et al. (2008) and implemented by Lavado (2018).

#### Parameter Computation

The parameters retrieved from the neurons were total dendritic length, apical dendritic length, basal dendritic length, and number and length of processes per category (primary, secondary, or higher). To obtain those parameters, a single segment was manually identified as the soma, as to orient the graph generating a tree-like structure. To define apical/basal length, a single process starting from the soma, which contains the segments defined as the apical reference, defined the apical process. To identify primary processes, the longest paths of each of the segments starting from the soma were computed, and recursively, the same algorithm repeated along the branches to identify higher order processes.

### Slice Preparation and Extracellular Field Recording in Auditory Cortical Synapses

To prepare brain slices, animals were quickly decapitated and brains removed, immersed in ice-cold dissection buffer (in mM; 212.7 sucrose; 5 KCl; 1.25 NaH_2_PO_4_; 3 MgSO_4_; 1 CaCl_2_; 26 NaHCO_3_; 10 glucose; pH 7.4), and coronal slices (400 µm thickness) were obtained using a vibratome (Ted Pella Inc., USA). Slices were transferred to an interface chamber containing artificial cerebrospinal fluid (aCSF) saturated with 95% O_2_–5% CO_2_ at 36°C, left at these conditions for 45 min, and then maintained at 24°C for 1 h.

Field excitatory postsynaptic potentials (fEPSP) recorded from auditory cortex were evoked as described in Hefti and Smith (Hefti and Smith, 2000). Briefly, electrical stimulation (biphasic, constant current, 200 ms stimuli) was delivered every 15 s to the layer I in the primary auditory cortex of control and fluoxetine-injected rat brain slices, using a concentric bipolar electrode (FHC Corporate and Manufacturing, Bowdoin, ME, USA) and recorded in layers II–III. Recording electrodes comprised glass micropipettes (1–3 MΩ) filled with aCSF. Baseline recordings were performed using oxygenated (5% CO_2_–95% O_2_) Mg^2+^-free aCSF at 32°C on the recording chamber continually perfused at a flow of 2 ml/min. After 10 min, the recording solution was replaced by Mg^2+^-free aCSF containing 20 µM 6-cyano-7-nitroquinoxaline-2,3-dione (CNQX) and 10 µM Picrotoxin, to block α-amino-3-hydroxy-5-methyl-4-isoxazolepropionic acid (AMPA) and gamma-aminobutyric acid receptors (GABA_A_) receptors. After incubation of 20 min, the NMDA-R-mediated component of the fEPSP was recorded for 10 additional minutes. Finally, the recording chamber solution was replaced a second time by an aCSF containing 20 µM CNQX, 10 µM Picrotoxin, and 150 nM ifenprodil, to block GluN2B-containing NMDA receptors, and incubated again for 20 min to record the remaining GluN2A-mediated NMDA-R component for 10 additional minutes. Data recorded from pharmacological experiments were acquired using an extracellular amplifier (Model EX 4-400 Differential Extracellular Amplifier, Dagan Corporation, USA) and a data acquisition board (National Instruments, USA) controlled through Igor Pro software (WaveMetrics Inc, Lake Oswego, Oregon, USA). All the electrophysiological experiments were performed using tissues from at least eight different animals for each condition.

### Analysis of Field Excitatory Postsynaptic Potentials Obtained From the Auditory Cortex

To determine differences between fEPSP obtained from control and fluoxetine-treated slices, acquired data were transferred to the scientific graphing and data analysis software Origin 9.0. Thereafter, the rising and decay portions of each curve were independently adjusted to a Boltzmann curve, obtaining from the fitted rising part the peak and rise time parameters, whereas decay time was obtained from the latter. The area under curve was calculated using the first 30 ms of the curve, taking as time 0 the beginning of the fEPSP curve.

### Statistical Analysis

The values are shown as the means ± SEM. Statistical significance was evaluated by Mann–Whitney *U*-test for morphological data (Golgi staining and 3D reconstruction) and two way-ANOVA followed by *post hoc* Bonferroni correction for electrophysiological data (GraphPad Prism software, San Diego, USA), as indicated in the figure legends. A probability level of 0.05 or less was considered significant.

## Results

### Repetitive Fluoxetine Administration Induces Shortening of the Dendritic Arbor of Pyramidal Neurons

**Figure 1:**
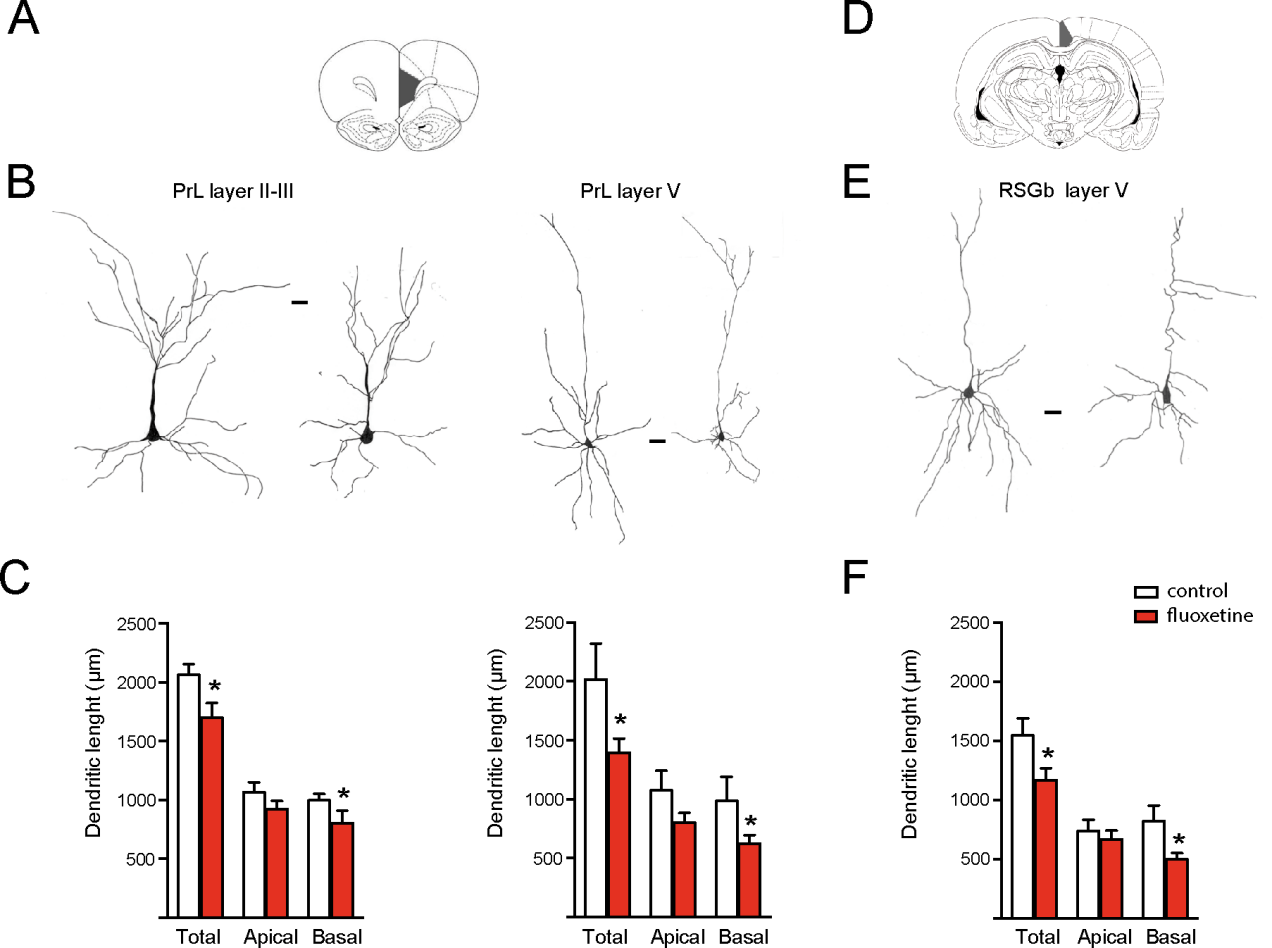
Chronic fluoxetine treatment (28 days) decreased the dendritic length of pyramidal neurons in limbic cortices. Golgi staining was performed to analyze the prelimbic (PrL) and retrosplenial granular b (RSGb) cortices. Neurons were drawn with camera lucida and digitally scanned to visualize the morphology. **(A)** and **(D)** The analyzed areas are schematically shown (Paxinos and Watson, 1998). **(B)** and **(E)** Representative drawing of pyramidal neurons obtained from vehicle treated (control) or fluoxetine-treated rats. In the PrL cortex, layer II–III and V neurons were analyzed, while in the RSGb cortex, the analysis was restricted to layer V neurons. In each case, the neuron at the left is from the control (vehicle treated), while the neuron at the right is from the fluoxetine-treated animal. Scale bar: 20 µm. **(C)** and **(F)** Quantifications of the total, apical, and basal dendritic lengths of neurons in the PrL cortex (layers II/III in the left panel and layer V in the right panel) and RSGb cortices. Values represent the mean ± SEM. For each condition, we analyzed at least 10 neurons obtained from four animals per condition for the PrL cortex and three animals per condition for the RSGb cortex. *p ≤ 0.05, Mann–Whitney *U*-test.

**Figure 2:**
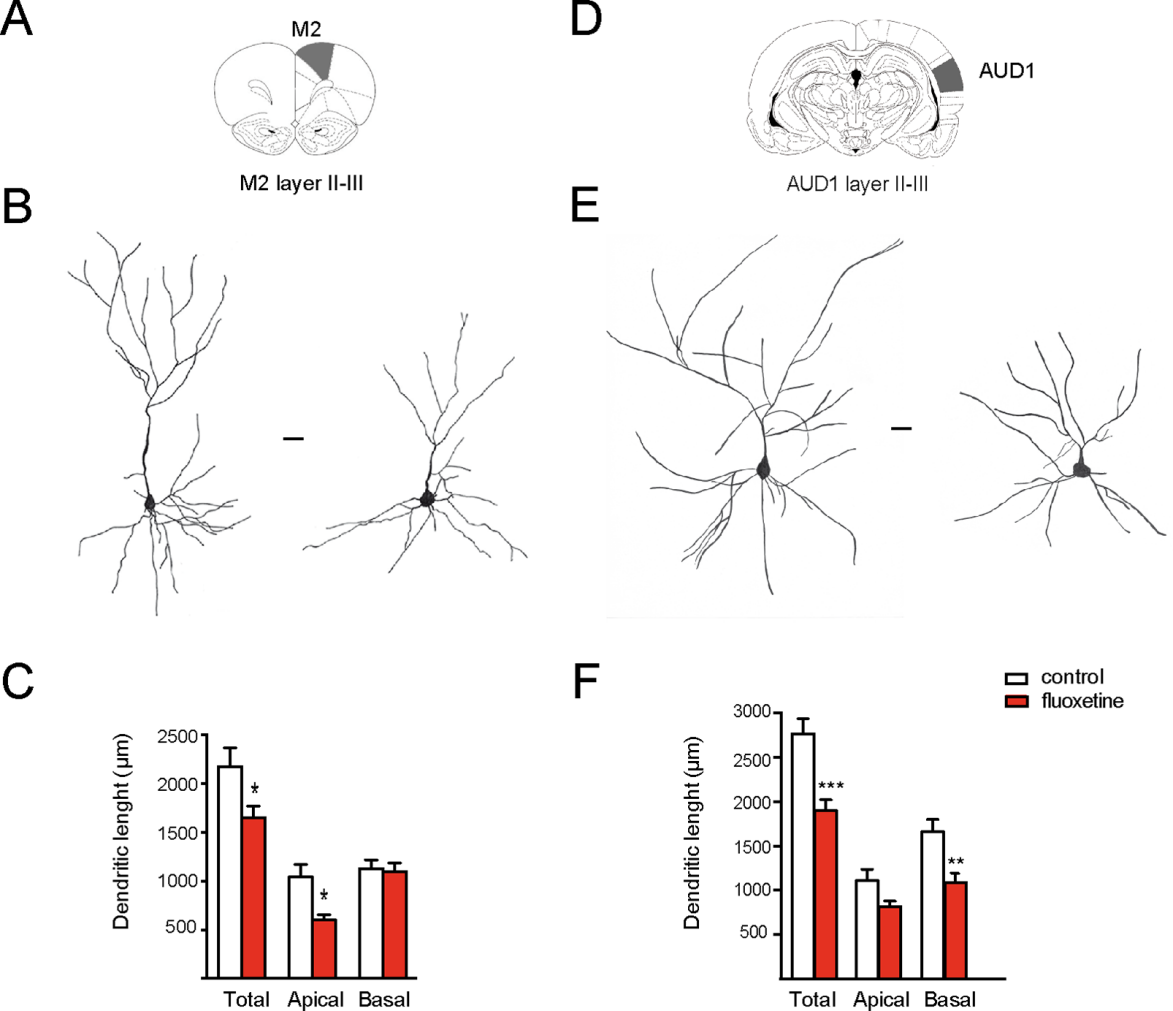
Chronic fluoxetine treatment (28 days) decreased the dendritic length of pyramidal neurons in the motor and auditory cortices (layer II–III). Golgi-stained neurons of the II–III layer of the secondary motor cortex (M2) and the primary auditory cortex (AUD1) were drawn with camera lucida and digitally scanned. **(A)** and **(D)** Scheme of the analyzed areas (Paxinos and Watson, 1998). **(B)** and **(E)** Representative drawings of neurons obtained from vehicle or fluoxetine-treated animals. In each case, the neuron at the left is from the control (vehicle-treated), while the neuron at the right is from the fluoxetine-treated animal. Scale bar: 20 µm. **(C)** and **(F)** Quantifications of the total, apical, and basal dendritic lengths. For each condition, we analyzed at least 10 neurons obtained from three animals per group (in the case of M2) and from three (control) and four (fluoxetine-treated) animals in the case of AUD1. Values represent mean ± SEM. *p ≤ 0.05; **p < 0.01 ***p < 0.001 Mann–Whitney *U*-test.

We had previously shown that repetitive fluoxetine treatment augmented the spine density and the number of mature, mushroom-type spines and favored GluN2A-containing NMDARs and GluA2-containing α-amino-3-hydroxy-5-methylisoxazole-4-propionate receptors (AMPA-Rs) (Ampuero et al., 2010; Ampuero et al., 2013; Rubio et al., 2013). Consistently, this switch in glutamate receptor subunits was associated with decreased calcium influx, synaptic plasticity, and hippocampus-dependent learning (Ampuero et al., 2013; Rubio et al., 2013; Beshara et al., 2016). Taking into consideration that glutamate receptor subunits determine dendritic morphology in neurons, we then examined morphological changes in dendritic length and complexity (Bustos et al., 2017; Keith et al., 2019). Using the Golgi staining method, we measured the dendritic length of apical and basal branches of pyramidal neurons in several cortical areas. In two limbic cortices, e.g., the prelimbic cortex (PrL) and retrosplenial granular b cortex (RSGb), a decrease in the total dendritic length after fluoxetine treatment was observed, and this resulted from a reduction in the basal dendritic lengths in layer II–III neurons of the PrL cortex (in µm, control = 998 ± 53; fluoxetine = 802 ± 108, *p* = 0.045) and in layer V neurons (in µm, control = 986 ± 204; fluoxetine = 624 ± 72, *p* = 0.038). Similarly, the basal dendritic length decreased in layer V neurons of the RSGb cortex (in µm, control = 821 ± 134; fluoxetine = 499 ± 54, *p* = 0.020) (**Figure 1** and **Supplementary Table 1**). In the RSGb, we did not obtain reliable staining of layer II–III neurons. Similarly, in cortical areas not directly involved in antidepressant action, e.g., pyramidal neurons of the II–III layer of the secondary motor cortex (M2) and primary auditory cortex (AUD1), a reduction in the dendritic length was shown: in M2, apical branches were reduced (in µm, control = 1,043 ± 129; fluoxetine = 598 ± 60, *p* = 0.014), while in AUD1, the basal dendritic length was affected (in µm, control = 1,661 ± 138; fluoxetine = 1,086 ± 99, *p* = 0.0025) (**Figure 2** and **Supplementary Table 1**).

**Figure 3:**
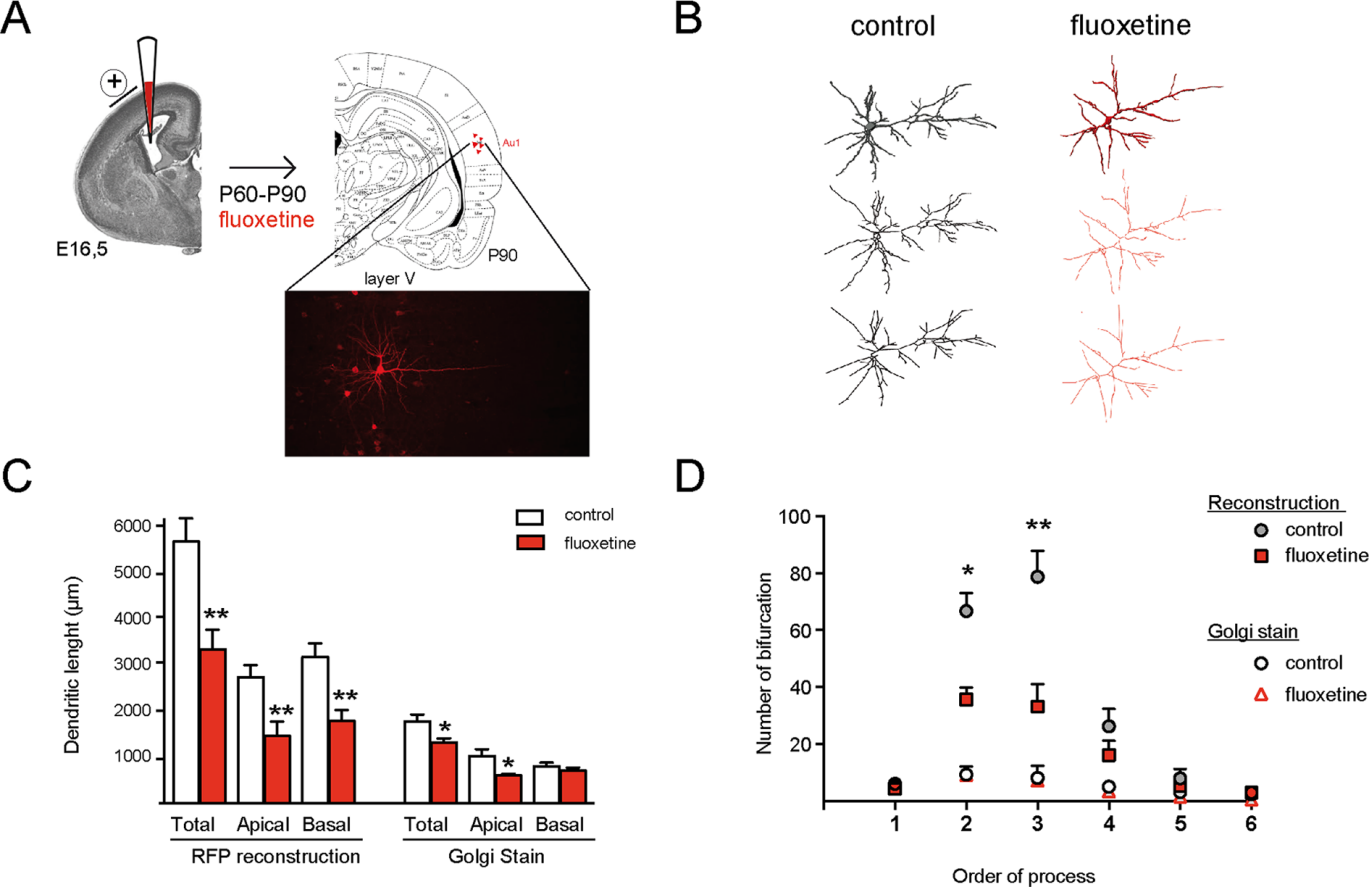
Repetitive flx treatment decreases the dendritic length of pyramidal neurons in the primary auditory cortex of rats. **(A)** Schematic representation of in utero electroporation to target gene delivery into neurons in the auditory cortex. The inset shows mRFP electroporated pyramidal neurons selected for analysis in layer V of the primary auditory cortex in coronal slices of 150 µm. **(B)** The 3-dimensional reconstruction of confocal images is shown for isolated neurons obtained from vehicle and fluoxetine-treated animals. Upper panel: raw image; middle panel: skeletonized form; and lower panel: the reduced skeletonized form. **(C)** Quantifications (mean ± SEM) of the total, apical, and basal dendrites, measured in the skeletonized form of neurons after 3-dimensional reconstruction (left grouped bars) or measured after Golgi staining of the same area (right grouped bars). **(D)** Quantification of the number of bifurcations according to increasing orders of processes. Value represent mean ± SEM. For 3-dimensional reconstruction, eight control neurons obtained from three animals and seven neurons obtained from three animals were analyzed. *p ≤ 0.05; **p < 0.01 Mann–Whitney *U*-test.

Although the Golgi method has been used for decades to assess dendritic arbor morphology, this technique does not allow a 3-dimensional reconstruction. We thus used mRFP expression in cortical neurons to analyze the dendritic arbor of pyramidal neurons from layer V of AUD1 (**Figure 3** and **Supplementary Table 2**). In **Figure 3A**, an overview of the electroporation procedure, including expression of the fluorescent protein in isolated neurons, is shown. In **Figure 3B**, a representative image of the 3-dimensional reconstructed branches of neurons obtained from control or repetitive fluoxetine-treated animals is shown. Representative images at different levels of analysis are indicated, showing the raw data in the upper panel, the skeletonized data in the middle panel, and the reduced skeleton in the lower panel. The analysis of the reduced skeleton revealed a significant reduction in both the apical (in µm, control = 2,739 ± 272; fluoxetine = 1,460 ± 315, *p* = 0.008) and the basal (in µm, control = 3,178 ± 302; fluoxetine = 1,786 ± 242, *p* = 0.008) dendritic length after fluoxetine (**Figure 3C**). When comparing both experimental approaches to assess the dendritic length, we found (as expected) greater dendritic length when the 3-dimensional reconstruction was performed, as well as a significant reduction of basal dendrites lengths after fluoxetine, in contrast to the Golgi method with which this difference was not significant and thus was not detectable with the 2-dimensional analysis. Similarly, decreased complexity of the dendritic tree was detected after fluoxetine when using the 3-dimensional method but not the Golgi method (**Figure 3D**). This resulted from a decrease in the number of secondary and tertiary processes, while primary processes were not affected. This result is consistent with the morphological consequences observed in neuronal cultures in which GluN2A/GluN2B ratios are manipulated (Bustos et al., 2014; Bustos et al., 2017). Now, we also show that repetitive fluoxetine treatment induces a general reduction in the dendritic arbor in limbic and nonlimbic cortical areas, which is indicative of increased maturation. These results are also consistent with previous observations of increased dendritic spine density and of mushroom-type synapses after fluoxetine (Ampuero et al., 2010; Rubio et al., 2013).

### Electrophysiological Recordings in the Auditory Cortex Suggest Increased Synaptic GluN2A Subunit Levels

**Figure 4:**
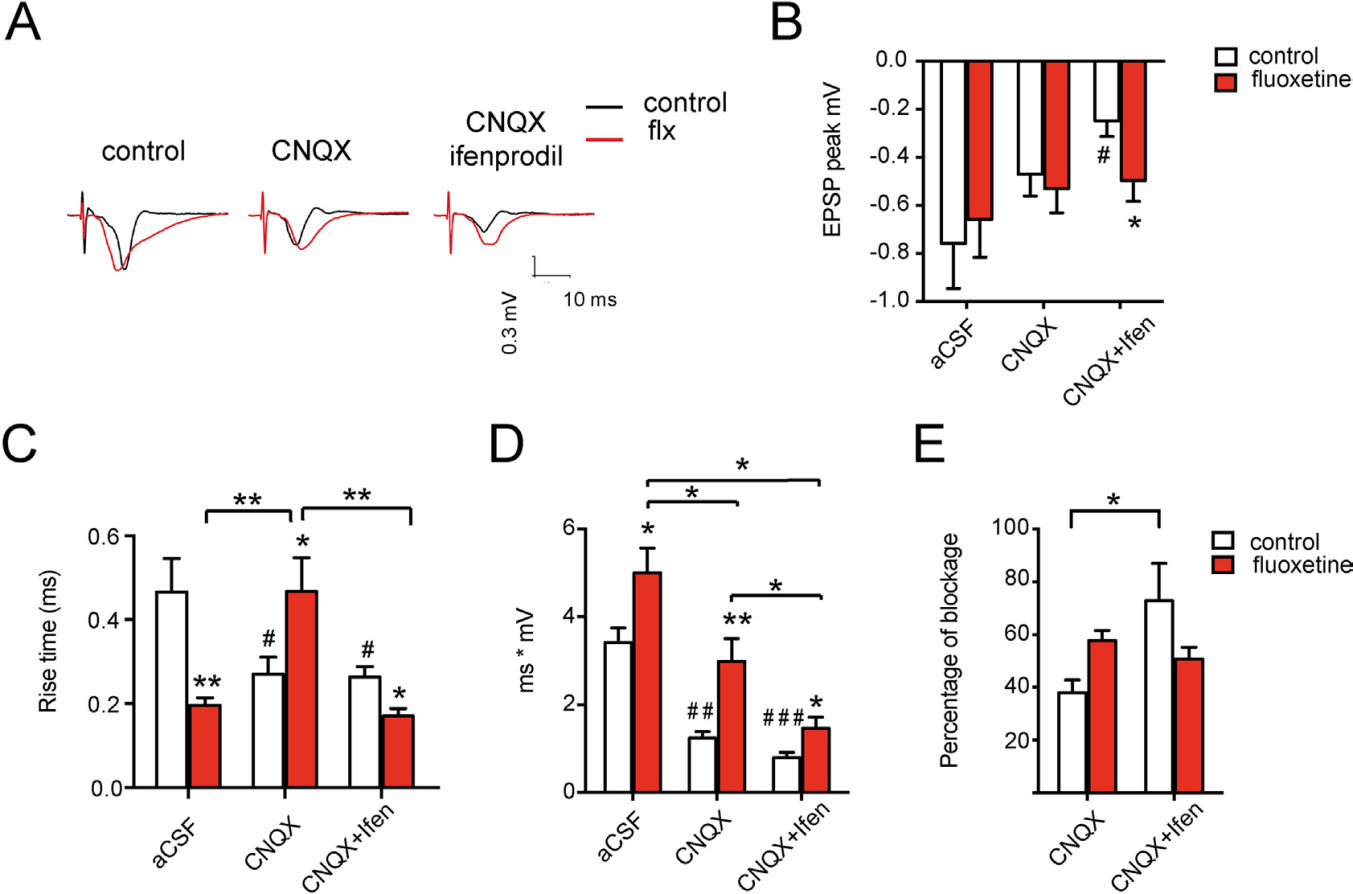
Chronic fluoxetine treatment modifies the postsynaptic response in the auditory cortex of rats. Different parameters of the fEPSPs were measured after stimulation in layer I and recording in layer II–III: **(A)** Representative recordings in slices from vehicle or fluoxetine-treated animals (left) followed by the NMDA-mediated response (in the presence of CNQX to block AMPA receptors) (middle) and of the GluN2A-mediated response (in the presence of CNQX + ifenprodil to block AMPA and GluN2B-containing receptors) (right). **(B)** Peak fEPSPs, **(C)** rise time (or the time at which the 67% of the peak potential is achieved), **(D)** total postsynaptic potential (area under the curve), and **(E)** percentage of blockade of the total fEPSP by CNQX (NMDA component) or by CNQX + ifenprodil (GluN2A component). Slices were perfused with Mg-free aCSF (control recording) or with aCSF in the presence of CNQX and then in the presence of CNQX + ifenprodil. For each condition, at least 32 slices were recorded, obtained from 16 animals (*n* = 8 control and *n* = 8 fluoxetine). Figures show the mean ± SEM. One way-ANOVA followed by *post hoc* Bonferroni test was used. *p ≤ 0.05; **p < 0.01 relative to the control within each drug condition (i.e., within each pair of red vs. white bar). For indicated comparisons among groups, unpaired *t*-tests were used ^#^ *p* ≤ 0.05; ^##^ *p* < 0.01, ^###^ *p* < 0.001 relative to the first bar (aCSF-control without drug addition).

To assess whether the evoked field potentials in the auditory cortex are consistent with increased synaptic GluN2A levels (Ampuero et al., 2010), we evaluated the NMDA-mediated responses by blocking AMPA receptors with CNQX. Additionally, we blocked GluN2B-containing NMDA-Rs with ifenprodil in a sequential manner, using slices obtained from control and fluoxetine-treated animals (**Supplementary Table 3**). **Figure 4A** shows representative traces of these experimental conditions, while in **Figure 4B–E**, different parameters of the fEPSP are evaluated. The peak amplitude of the total or NMDA-mediated response did not change (**Figure 4B**). However, ifenprodil was ineffective in reducing the peak amplitude of the field excitatory postsynaptic potential (fEPSP) after fluoxetine treatment (in mV, control = −0.247 ± 0.066; fluoxetine = −0.499 ± 0.087, *p* = 0.038 in the condition CNQX plus ifenprodil), consistent with a GluN2B-to-GluN2A switch. In the same line, control responses were blocked by CNQX plus ifenprodil (in mV, aCSF = −0.758 ± 0.019; CNQX + ifen = −0.247 ± 0.066; *p* = 0.017), while in slices of fluoxetine-treated rats, CNQX plus ifenprodil did not block the response with respect to aCSF. Moreover, **Figure 4C** shows a significant decrease in the rise time when the AMPA and GluN2B components of the fEPSP were removed (i.e., in the presence of CNQX plus ifenprodil) (in ms, control = 0.264 ± 0.02; fluoxetine = 0.17 ± 0.02, *p* = 0.008), consistent with an increase in GluN2A receptor subtypes, which are known to present faster rise time kinetics than other GluN2 subunits (Hansen et al., 2018). Interestingly, the integrated response over 30 ms (area under the curve, mV*ms) increased in all conditions after fluoxetine compared to control animals (**Figure 4D**). In this case, the total aCSF response was control = 3.4 ± 0.3; fluoxetine = 5.0 ± 0.6 (*p* = 0.029); the NMDA-mediated response after AMPA-R blockade was of control = 1.2 ± 0.2; fluoxetine = 3.0 ± 0.5 (*p* = 0.029); and the GluN2A-mediated response was of control = 0.80 ± 0.1; fluoxetine = 1.5 ± 0.3 (*p* = 0.031). These results are consistent with previous data showing a larger proportion of mushroom-like spines and increased spine density after fluoxetine administration (Ampuero et al., 2010). In addition, the percentage of blockade of the integrated NMDA-mediated response (i.e., in the presence of CNQX) after ifenprodil decreased in treated animals, revealing a relative reduction in the GluN2B-mediated response (**Figure 4E**). The following percentages of blockade were observed: in control slices, the blockade by CNQX was of 37.93 ± 4.9% and increased to 72.86 ± 14% when ifenprodil was added (*p* = 0.035). In contrast, in slices from fluoxetine-treated rats, blockade by CNQX was 57.68 ± 3.8%, and after ifenprodil, it was only 50.79 ± 4.5%, revealing (as in **Figure 4B**) a failure of ifenprodil to block the synaptic response; this is consistent with a reduction in the GluN2B-mediated response after fluoxetine treatment.

## Discussion

The morphological and electrophysiological data obtained in the present work show that the treatment of adult rats with fluoxetine during 4 weeks induced a reduction in the dendritic arbor in cortical pyramidal neurons and a gain of GluN2A over GluN2B mediated fEPSPs in the auditory cortex. The 3-dimensional reconstruction of neurons from serial microscopic images provided an advantageous and powerful strategy for better visualizing changes in dendritic morphology. We propose that the fluoxetine-induced sculpting of neuronal circuits could be useful in the treatment of brain disorders characterized by a shift towards pathologically increased neuronal sprouting and altered synaptic transmission, such as in some forms of epilepsy.

### Morphological Changes in Pyramidal Neurons After Fluoxetine

We and several other groups had previously reported that fluoxetine increased spine density as well as the proportion of mushroom-type (i.e., mature) spines in several forebrain regions (Hajszan et al., 2005; Guirado et al., 2009; Ampuero et al., 2010; Kitahara et al., 2016). A spine increment in the somatosensory cortex with the use of the rapid acting antidepressant ketamine has been proposed as a common and necessary mechanism involved in effective antidepressant treatment (Pryazhnikov et al., 2018). However, the functional consequences of such changes for neuronal network activity remain uncertain. In our experiments, we also observed increased spine density but decreased dendritic length. To estimate whether fluoxetine changes the total number of synapses, we calculated whether a mean increase of 21.4 ± 3.4% in spine density (Ampuero et al., 2010; Rubio et al., 2013) and a mean decrease in the dendritic length of 25.7 or 41.3% (Golgi method or 3-dimensional reconstruction, respectively) affects the total synapse number. With both estimations, it could be concluded that the number of total synapses does not change (Golgi method or 3-dimensional reconstruction, respectively). Note that as spine density varies along primary and higher order dendrites, these estimations are simple approximations based on the mean percentage of spine increase. However, as reported by us and by others, the synaptic contacts shift to a more stable type (i.e., mushroom type), putatively affecting in a positive manner the strength of certain synaptic connections but causing the pruning of others.

### Synaptic Responses in the Auditory Cortex

In patients, it has been shown that auditory cortex activation and auditory perception and processing are impaired in depression (Zwanzger et al., 2012; Bonetti et al., 2017; Zweerings et al., 2019). Moreover, age-related hearing loss has been associated with late life depression (Husain et al., 2014; Rutherford et al., 2018; Scinicariello et al., 2019), and in animals, chronic restraint stress decreased glucose metabolism in the auditory cortex (Wei et al., 2018). It would be relevant to know whether antidepressant medication reverts auditory dysfunction. In our study, we addressed the effects of fluoxetine in naïve (nondepressed) rats.

The electrophysiological recordings of fEPSPs in the auditory cortex were compatible with increased glutamatergic synaptic response and increased GluN2A levels in synapses (Ampuero et al., 2010; Rubio et al., 2013). In fact, the amplitude of the evoked and integrated response increased in all the examined conditions (total, NMDA-mediated and GluN2A-mediated), revealing that, independent of the postsynaptic glutamate receptor composition, the glutamatergic response increased. In addition, the ineffectiveness of ifenprodil in reducing the NMDA-mediated response after fluoxetine administration indicated a loss in the contribution of GluN2B receptors to synaptic fEPSPs, and the fEPSP rise time decreased when the GluN2B component was removed with ifenprodil, unmasking in such a way the characteristic GluN2A fast rise time kinetics, as reported previously (Amico-Ruvio et al., 2012).

Consistent with the idea of more mature and less plastic synapses at AUD1 intracortical synapses, the field postsynaptic potential augmented after fluoxetine while long-term potentiation (LTP) was impaired (Dringenberg et al., 2014). Similar effects of fluoxetine were observed by us and others in the hippocampal CA1 region (Rubio et al., 2013; Van Dyke et al., 2019). These changes, indicating more mature but less plastic synapses, were corroborated using glutamatergic markers which also extended to GABAergic markers of synaptic maturation in the visual cortex (Beshara et al., 2016). We postulate that the plasticity-inducing effect of fluoxetine described by others (Maya Vetencourt et al., 2008; Kobayashi et al., 2010; Guirado et al., 2014; Ruiz-Perera et al., 2015) might be compatible, at different time points, with the synaptic stabilization and dendritic pruning observed in our rat model. However, it is not yet known which temporal changes during the treatment period may sequentially induce a plasticity period, followed by a stabilization period characterized by a “mature” network. The time course of these changes might be related to region-specific differences in serotonin receptor expression types and/or levels (Charnay and Leger, 2010). Auditory processing is impaired in major depression, and this is reverted by SSRIs (Frodl, 2017). We have focused here on the auditory cortex because the participation of serotonin in the auditory processing is well documented, and it can be modulated after repetitive administration of the SSRI fluoxetine (Ji and Suga, 2007; Hurley and Hall, 2011).

### Therapeutic Potential Associated to Selective Reduction of GluN2B Subunits and of Dendritic Tree Length

Our results indicate that the SSRI fluoxetine induced dendritic and synaptic pruning in areas of the cerebral cortex not directly implicated in the main symptoms of depression. In principle, these pro-maturational effects could be useful for restoring stable synaptic networks in pathologies where a shift towards immature circuits and/or to GluN2B-dependent neurotoxicity occurs. Thus, in animal models of temporal lobe epilepsy, the GluN2B/GluN2A ratio increased in several forebrain regions (Amakhin et al., 2017; Zubareva et al., 2018), and synapses enriched in GluN2B subunits favored seizure susceptibility (Okuda et al., 2017), whereas blockade of GluN2B receptors during experimental status epilepticus with compounds that included the rapid acting antidepressant ketamine (a non-selective NMDA-R antagonist) reduced neuronal damage (Loss et al., 2019). Moreover, fluoxetine and other SSRIs are considered to be safe in the treatment of depression in epileptic patients, although exceptions (e.g., in some epilepsy subtypes) to this general rule might exist (Kanner, 2016a; Kanner, 2016b). However, the use of fluoxetine to favor GluN2A-associated structural and functional synaptic changes in nondepressed epileptic patients or even in preventing oxidative stress associated to neurological disorders is open to discussion and additional experimental work (Zhu et al., 2018).

Interestingly, the neuroprotective potential of direct and selective GluN2B blockade has extensively been addressed in several brain disorders (Gielen et al., 2009; Mony et al., 2009). Moreover, uncoupling of GluN2B C-terminal-dependent signaling or selective GluN2B blockade has antidepressant effects (Li et al., 2018) and might exert neuroprotective properties in Alzheimer’s disease, Huntington’s disease, and after stroke (Dau et al., 2014; Wu and Tymianski, 2018; Xu et al., 2019). In all these cases, fluoxetine could complement therapeutic strategies aimed at preventing or reducing neurotoxicity. In additional observations, fluoxetine reduces β-amyloid levels and toxicity, an effect that may depend on subunit-specific NMDA-R blockade and increased GluN2A-dependent brain-derived neurotrophic factor (BDNF) and transforming-growth-factor-β1 (TGF-β1) signaling (Tucci et al., 2014; Schiavone et al., 2017; Caraci et al., 2018). Moreover, fluoxetine displays direct GluN2B antagonism, an effect that results convergent with the GluN2B to GluN2A subunit switch and the morphological consequences described by us (Kiss et al., 2012). This selectivity would be advantageous over the nonselective NMDA-R antagonist ketamine, a promising novel antidepressant drug with unwanted side effects that have yet to be overcome (Duman, 2018; Orhurhu et al., 2019). Interestingly, several mechanisms underlying the therapeutic action of ketamine are shared with fluoxetine, but with a different time scales. In such a way, GluN2B subunits are essential for the antidepressant effect of ketamine and the downstream activation of mTOR signaling (Wang et al., 2011; Miller et al., 2014) and ketamine regulates positively spinogenesis (Moda-Sava et al., 2019).

Taken together, the morphological changes observed by us after repetitive fluoxetine administration, consistent with a GluN2B subunit reduction in several forebrain areas, may have promising applications in brain disorders beyond depression.

## Author Contributions

EA and UW designed experiments and wrote the manuscript; AG participated and instructed in *in utero* electroporation experiments and revised the manuscript; MC, EA, and SH did 3-dimensional reconstruction and quantifications; FR and SM did *in utero* electroporation and drug treatments; PC, EA, and LA-C did Golgi staining and analysis blind to the experimental condition; FR and RS critically reviewed the manuscript; and RS did electrophysiological recordings.

## Funding

This work was supported by Fondecyt 1140108 (to UW), 1181823 (to SH) and 11161033 (to MC), Conicyt (to EA), CONICYT PIA ACT1402, ICM P09-015-F, and DAAD 57220037 and 57168868 (to SH and MC), and CORFO 16CTTS-66390 (to SH).

## Conflict of Interest Statement

The authors declare that the research was conducted in the absence of any commercial or financial relationships that could be construed as a potential conflict of interest.
