# Supplementary figures and images for: Chronic Fluoxetine Treatment Induces Maturation-Compatible Changes in the Dendritic Arbor and in Synaptic Responses in the Auditory Cortex
Source: Front Pharmacol. 2019 Jul 17;10:804. doi: 10.3389/fphar.2019.00804 (PMC6650542; doi:10.3389/fphar.2019.00804)

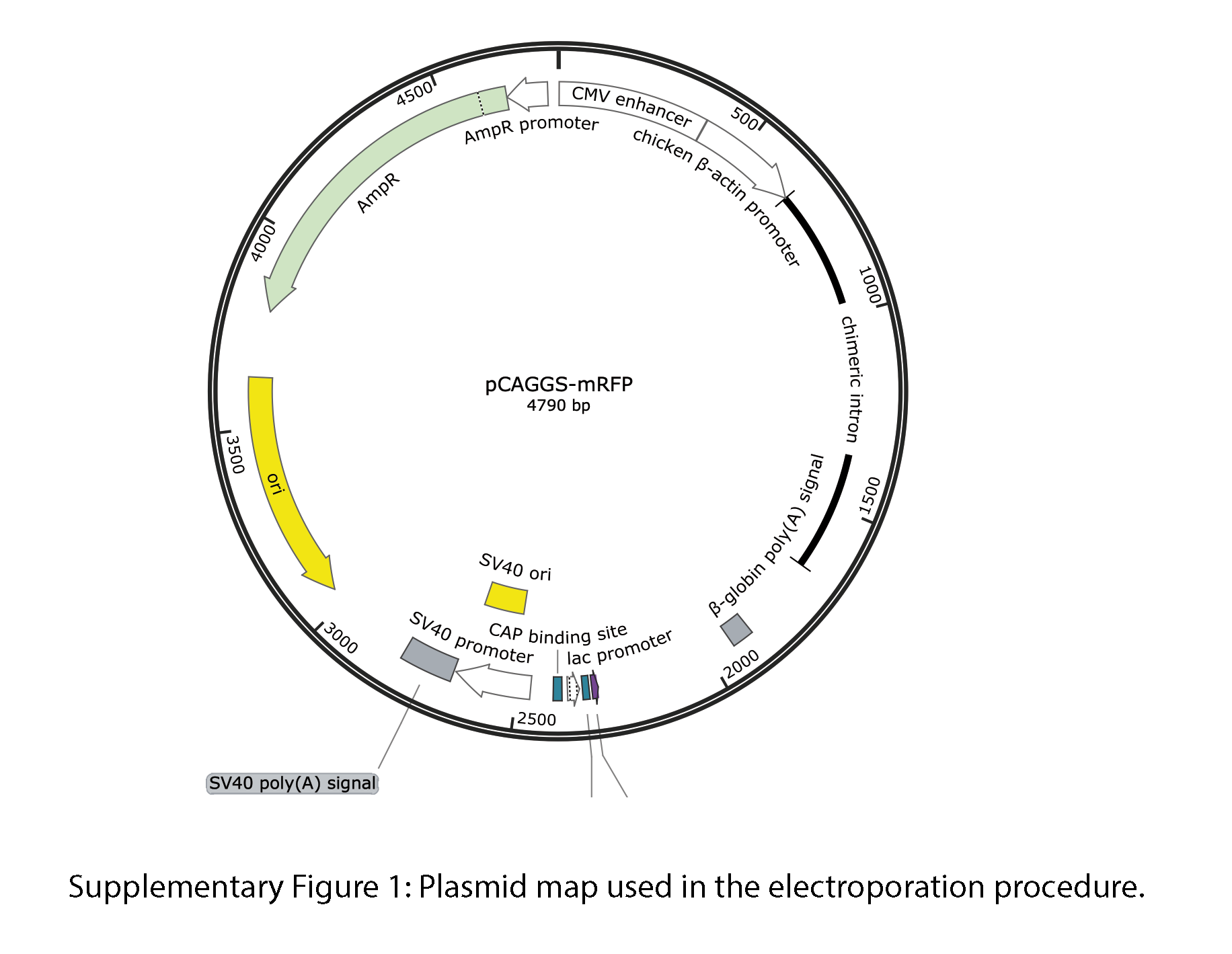

Supplement: Supplementary file 1 [file Image_1.tif]

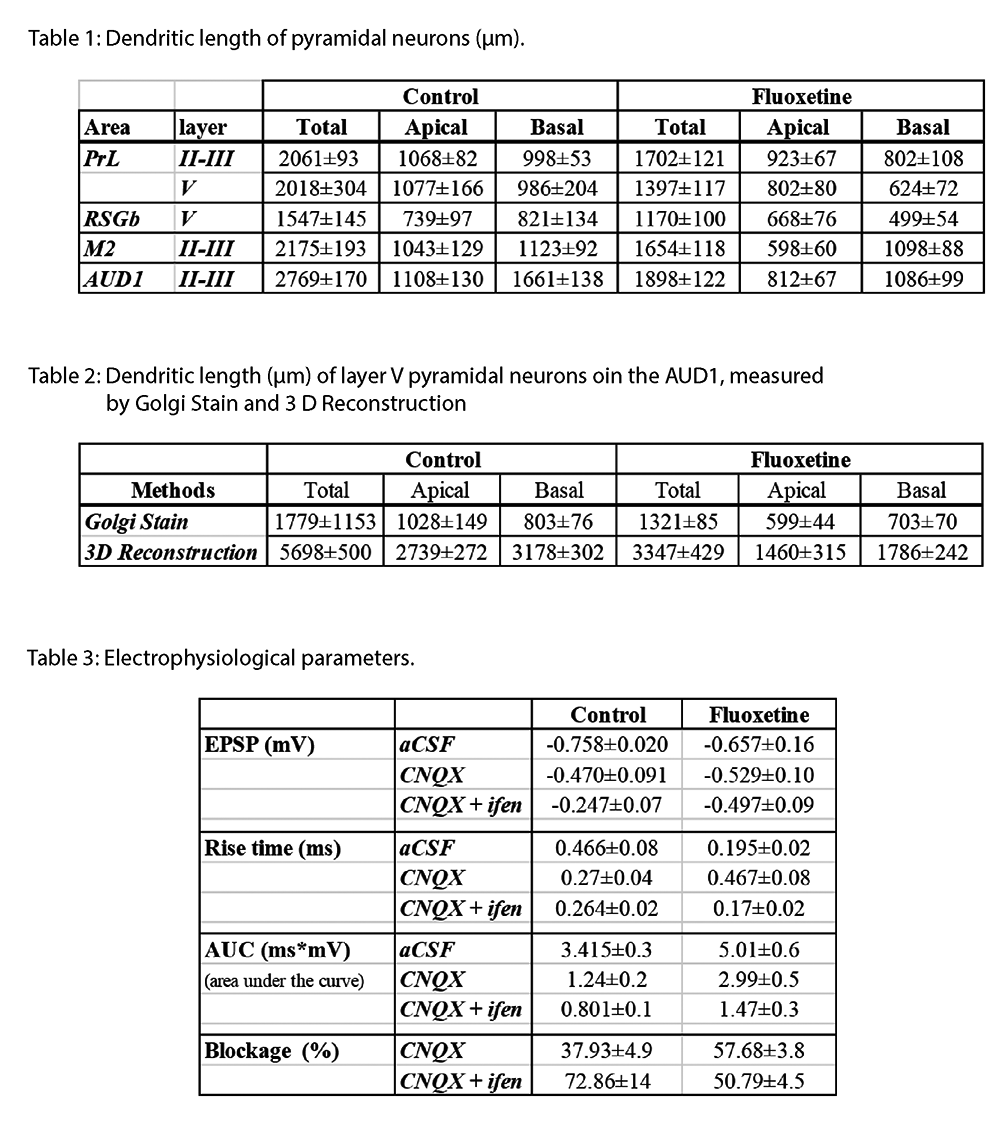

Supplement: Supplementary file 2 [file Image_2.tif]
